# Identification of Genomic Regions for Carcass Quality Traits within the American Simmental Association Carcass Merit Program

**DOI:** 10.3390/ani11020471

**Published:** 2021-02-10

**Authors:** Jordan K. Hieber, Rachel L. Endecott, Jane A. Boles, Jennifer M. Thomson

**Affiliations:** 1 Department of Animal and Range Sciences, Montana State University, Bozeman, MT 59718, USA; jordan.k.hieber@gmail.com (J.K.H.); jboles@montana.edu (J.A.B.); 2 Grey Horse Consulting, McAllister, MT 59740, USA; rachel@greyhorseconsultingllc.com

**Keywords:** carcass weight, marbling, back fat, genetic correlation, genome-wide association

## Abstract

**Simple Summary:**

The American Simmental Association has an ongoing project assessing carcass composition and quality. Typically, when animals for mating are selected on maternal traits, there is a concomitant reduction in carcass value as many maternal traits are antagonistic to carcass traits. Analysis of the current data showed the correlation between hot carcass weight and calving ease direct to be lower than previously reported. This shows that multi-trait selection reduced the problem that arose when selecting for carcass traits or maternal traits individually.

**Abstract:**

USDA quality and yield grade are primary driving forces for carcass value in the United States. Carcass improvements can be achieved by making selection decisions based on the results of genetic evaluations in the form of expected progeny differences (EPD), real-time ultrasound imaging, and physical evaluation of candidate breeding animals. In an effort to advance their ability to accurately predict the breeding value of potential sires for carcass traits, the American Simmental Association launched the Carcass Merit Program as a means to collect progeny sire group carcass information. All records were extracted from the American Simmental Association database. Progeny data were organized by sire family and progeny performance phenotypes were constructed. Sire genotypes were filtered, and a multi-locus mixed linear model was used to perform an association analysis on the genotype data, while correcting for cryptic relatedness and pedigree structure. Three chromosomes were found to have genome-wide significance and this conservative approach identified putative QTL in those regions. Three hundred ninety-three novel regions were identified across all traits, as well as 290 novel positional candidate genes. Correlations between carcass characteristics and maternal traits were less unfavorable than those previously reported.

## 1. Introduction

With the first matings at the Sheek Ranch in Cabool, Missouri, in the spring of 1997, the American Simmental Association (ASA) launched a program that has influenced all producers and users of Simmental genetics. Simply known as the Carcass Merit Project (CMP), the enrolled sires of the Simmental and Simbrah breeds have been randomly mated to 35,000 commercial females to collect difficult-to-get progeny carcass information by sire group. Ten thousand carcass records have been collected and carcasses average 74% USDA Choice quality grade and a 2.8 USDA carcass yield grade, with tenderness data having been completed. In terms of calving ease (CE), 4400 British heifers have been mated and the resulting calves have an average birth weight (BW) of 78.6 lbs. For feed conversion, 1318 sire-identified SimAngus™ (American Simmental Association, Bozeman, MT, USA) progeny steers have been used, resulting in an average dry matter feed conversion 6.3:1 and an average daily gain of 1.64 kg/day. Data have been collected from 41 different herds across 18 states, with steers fed and harvested in nearly every cattle feeding region in the United States. These data include all of the crossbred offspring as well as Simmental offspring. To clarify, the commercial dams included in analysis were 3.48% Angus, 0.03% Gelbvieh, 0.23% Red Angus, 70.23% Simmental, and 26.03% with no breed information available. The calves were 3.07% Angus, 0.03% Gelbvieh, 0.21% Red Angus, and 96.7% Simmental.

These data allowed the ASA to improve the accuracy of expected progeny differences (EPD) used for the selection of economically relevant traits. It improved confidence levels of traits, from calving assistance all the way to predicting end-point product value. In the last 25 years, a shift has occurred in the US beef industry from a commodity-based market to one that is based on quality or product yield [1,2]. This has been facilitated by increased accuracy of carcass merit EPD. In an effort to reduce product variability and improve profitability, many producers have added marbling (MARB) and carcass yield as selection criteria. This means that a large number of the current cow herds have females that possess merit in the area of MARB or carcass quality. This selection could result in changes in other traits. Previously, researchers have reported negative genetic correlations between maternal performance traits and carcass merit [3,4]. As a result, there is interest in how the current carcass-based selection impacts maternal performance and index-based selection criteria. There is still genetic variation that cannot be predicted with current genomic tools. Thus, the objective of this study was to identify genetic markers and quantitative trait loci (QTL) for carcass traits and to evaluate the correlations between carcass merit traits and maternal performance.

## 2. Materials and Methods

Data were gathered by the ASA CMP. All steer performance records containing calf BW, calving ease direct (CED) scores [5], calving ease maternal (CEM) scores, 205-day adjusted weaning weight (WW), 12th rib fat (BF), ribeye area (REA), MARB, hot carcass weight (HCW), and internal fat (KPH) were extracted from the ASA database. Sire EPD, performance, and single nucleotide polymorphism (SNP) 50 K genotype data were also obtained. The data were shared under a material transfer agreement between Montana State University and the American Simmental Association and are not publicly available. Dam EPD and performance data, if available, were also collected for the dam associated with each steer record.

Progeny data were organized into 150 sire families. Performance averages for all traits (*n* = 1–150 progeny per family) were calculated. Sires with either SNP50K or comparable imputed 50 K data from higher or lower density SNP chips were used in the overall analysis. Genotype data quality control was done through SNP filtering [6]. First, samples were removed with call rates ≤0.95, indicating reduced DNA quality. Next, markers were removed if: a call rate was <0.95, had >2 alleles, and a minor allele frequency (MAF) <0.01. Data were then filtered to remove markers in linkage disequilibrium with each other, leaving a marker to represent groups in linkage with each other and markers located on non-autosomal chromosomes. This left 37,552 out of 52,584 markers for further analysis.

Samples were then filtered to determine relatedness. An identity by state (IBS) relationship matrix was created to correct association analysis for genomic relationships amongst samples. This was done by utilizing the Identity by Descent (IBD) estimation tool within Golden Helix. The complete algorithm used by Golden Helix SVS is spelled out in Purcell et al. [7].

Principal component analysis (PCA) was used to account for cryptic relatedness and the first three eigenvectors represented greater than 50% of the stratification in the SNP data. Identity by Descent calculated relatedness between individuals and the first ten PCA-generated eigenvectors were used as covariates in the association analysis to account for pedigree structure and cryptic relatedness. A multi-locus mixed linear model (MLMM) [8] in Golden Helix SVS software (Golden Helix, version 8.7.2-2017-08-11) was used to perform regression-based association analyses on the genotype data. This method is an implementation of the MLMM method by Segura et al. [8] which uses a forward and backward stepwise approach to select markers as fixed effect covariates in the model and a pre-computed relationship matrix described above.

Benjamini–Hochberg multiple comparison corrections were used to minimize false-positive associations [9]. A genome-wide significance level utilizing the Benjamini–Hochberg correction with −log10(Adj. *p*-value) was 5 × 10^−8^ [10] and markers above the level of significance were used to identify regions of the genome associated with the trait in question. Regions with clusters of significantly associated markers were then labeled as putative QTL or genomic regions of interest and used to identify potential positional candidate genes. Additionally, suggested regions of interest were identified with a −log10(Adj. *p*-value) of 5 × 10^−5^ threshold, and these additional regions were also used to identify additional potential candidate genes for further research.

Genomic regions within 100,000 base pair (bp) of each significant marker were identified and highlighted. The same process was done for all of the identified markers for each chromosome, which created some overlap of highlighted areas. Previously reported significant markers were then cross-referenced with the highlighted areas. For significant markers (Adj. *p* < 0.001), the proportion of genetic variation explained by the marker was reported. This was determined by partitioning the variance for the model. This was re-estimated in each step of the MLMM, as described by Segura et al. [8]. 

To better understand how carcass-based selection impacts maternal performance, genetic correlations between each steer carcass trait were correlated to each of the maternal traits. Genetic correlations were estimated using the genomic best linear unbiased prediction (GBLUP) method to perform a bivariate restricted maximum likelihood (REML) analysis in SVS software (Golden Helix, version 8.7.2-2017-08-11). The following parameters were set for the analysis: residual covariance was not excluded, a gender correction was not applied as we used datasets of all dams for maternal data and all steer performance data, samples with missing phenotypes were dropped and we did not account for gene by environmental effects as we did not have that sufficient data to include it, and an IBD genomic relationship matrix was used for relatedness. These correlations were calculated using 1,681 animals that had either genotype and progeny data or genotype and individual data and 37,468 filtered markers.

## 3. Results

The dataset consisted of samples from 3849 individuals. Samples were grouped by sire and 2745 individuals had known sires, producing 395 sire families. Sire families ranged in size from one to 150 progeny (mean of overall family size = 6.88) with reported data for carcass traits. Progeny performance averages were constructed. The resulting 395 progeny performance averages were used in our association analysis which provided a smaller than ideal dataset for this purpose. As our primary objectives were to examine genetic correlations in this population and identify genomic regions of interest for further research, we proceeded, but we acknowledge the limitations of this dataset for identifying QTL and causative mutations with the smaller sample size represented.

**Figure 1:**
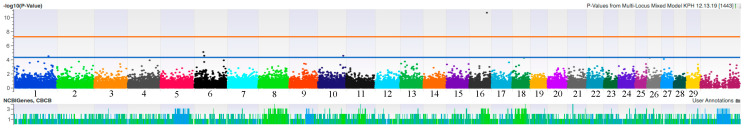
Manhattan plot for internal fat (KPH). Markers above −log10(*p*-value) of 5 × 10^−8^ (red line) are significant genome-wide association markers. Vertical clusters of markers are also of interest as they indicate potential quantitative trait loci (QTL) in those regions. Markers above −log10(*p*-value) of 5 × 10^−5^ (blue line) used to identify potential candidate genes for further research.

Genome-wide analysis found only one region for each: KPH (on chromosome 16, Figure 1), progeny average HCW (chromosome 20, Supplementary Figure S2), average MARB (chromosome 16, Supplementary Figure S4), and average BF (chromosome 17, Supplementary Figure S6), respectively, explained 0.0262, 0.0231, 0.023, and 0.0243% of the phenotypic variation (Table 1). 

Although the sire family structure of the data limited the resolution of our association analyses, areas of the chromosomes with vertical clusters of markers were of interest as they suggested putative QTL in those regions. Within a 100,000 bp window of each putative QTL region, positional candidate genes were identified using Genome Build Bos taurus ARS-UCD1.2. Putative QTL regions were compared to known QTL using AnimalQTLdb (http://www.animalgenome.org (accessed on 16 July 2020)). Previously reported QTL for carcass traits that overlap regions identified in this study are included in Supplementary Tables S1–S9 [11,12,13,14,15,16,17,18,19,20,21,22,23,24,25,26,27,28,29,30].

In addition to these previously identified regions, 393 novel regions were identified across all traits: 28 for HCW, 34 for MARB, 38 for BF, 35 for REA, 35 for KPH, 56 for average HCW, 39 for average MARB, 65 for average BF, and 63 for average REA. Of the positional candidate genes, four genes had been previously identified in other breeds. In addition to these, 290 novel positional candidate genes were identified across all traits (Supplementary Tables S1–S9).

Table 2 shows the correlations between each pair of traits in our dataset. These correlations were calculated using 1,681 animals that had either genotype and progeny data or genotype and individual data and 37,468 filtered markers. The objective was to evaluate the relationship between offspring carcass traits and maternal traits. The strongest negative correlations found in the dataset between carcass traits and maternal traits were between HCW and CED (−0.20), WW and milk (−0.18), and WW and CEM (−0.17).

## 4. Discussion

Carcass merit trait QTL have been identified in both US and Chinese beef cattle breeds. In a recent study by Saatchi et al. [18], 10 US beef breeds, including Simmental, were used to identify several QTL that overlapped with the significant regions in our study, including chromosome 6 for HCW and average BF. This demonstrates that our methods were able to generate similar results to those of a larger multi-breed analysis for the same traits.

In terms of positional candidate genes, Gill et al. [14] identified protein kinase AMP-activated non-catalytic subunit gamma 3 (*PRKAG3*) on chromosome 2 for HCW in Aberdeen Angus-sired steers. For MARB, retinoic acid receptor-related orphan receptor C (*RORC*) was identified on chromosome 3 by Barendse et al. [22] in Australian Angus, Brahman, and Hereford and by Barendse et al. [21] in Angus, Shorthorn, and other taurine breeds. For BF on chromosome 1, Kim et al. [20] identified interferon alpha and beta receptor subunit 1 (*IFNAR1*) in Angus–Brahman cross cattle and, on chromosome 5, Ujan et al. [28] identified myogenic factor 5 (*MYF5*) in Chinese *Bos taurus.* These results are similar to the results of our analysis and lend confidence to the regions and candidate genes we identified.

The correlation between HCW and CED (−0.20) was less than the correlation previously reported (−0.31) by MacNeil et al. [3] in crossbred steers and heifers. Likewise, the magnitude of previously reported negative correlations between REA, BF, and KPH and maternal traits are no longer as great, indicating reduction in the detrimental effects of selection for these disparate traits. Crews and Kemp [4] found a correlation of −0.23 for REA and BW in crossbred steers and heifers compared to the correlation of 0.10 found in this study. In crossbred steers and heifers, for BF and CED, MacNeil et al. [3] found a correlation of −0.36 and Splan et al. [31] found a correlation of −0.14 compared to the correlation of 0.002 found in this study. Splan et al. [31] also found a correlation of −0.29 for KPH and CED compared to the correlation of −0.01 found in this study. This indicates that multi-trait selection has been successful at decreasing the negative correlations between carcass characteristics and maternal traits in this population. The use of multi-trait selection to simultaneously improve two traits with a negative genetic correlation has been shown in plants [32,33] and in dairy cattle [34] in recent years and occurs when selective pressure is applied to both traits using an optimized approach or a selection index. Additionally we did not find the expected negative genetic correlations between docility and performance traits and this may be due to strong selection for docility in the Simmental breed over the last 20 years and that the variation in docility is less evident in this population.

## 5. Conclusions

Three chromosomes harboring QTL for various carcass traits were identified. Across all traits, 393 novel regions and 290 novel positional candidate genes were identified for the multi-locus model. Additionally, the detrimental genetic correlations between carcass characteristics and maternal traits are less than what has been previously reported, indicating that multi-trait- or index-based selection has been effective in reducing the strength of negative genetic relationships between traits. Further research is needed into the identified potential candidate genes and the role they may play in improving the value of beef carcasses. Additionally, this research supports the hypothesis that balanced selection on both maternal and carcass quality traits can improve productive efficiency. 

## Figures and Tables

**Table 1:** Markers identified to be significant in our genome-wide association analysis.

Trait ^1^	Chromosome	Base Pair Position	Proportion of Variation Explained
KPH	16	65,669,824	0.0262
Average ^2^ HCW	20	9,651,103	0.0231
Average ^2^ MARB	16	65,669,824	0.0230
Average ^2^ BF	17	46,357,742	0.0243

^1^ HCW = hot carcass weight; MARB = marbling; BF = 12th rib fat; KPH = internal fat. ^2^ Average denotes traits from constructed progeny performance averages.

**Table 2:** Genetic correlations and standard errors between carcass and maternal traits ^1^.

	MARB	BF	REA	KPH	CED	BW	WW	YW	CEM	Milk	MWW	Stay	Doc
HCW	0.53 (0.04)	0.40 (0.05)	0.63 (0.05)	0.37 (0.06)	−0.20 (0.03)	0.22 (0.04)	0.33 (0.03)	0.36 (0.03)	−0.04 (0.03)	−0.06 (0.04)	0.22 (0.03)	−0.04 (0.04)	0.03 (0.05)
MARB		0.35 (0.06)	0.28 (0.06)	0.52 (0.05)	0.07 (0.03)	−0.07 (0.04)	−0.10 (0.04)	−0.07 (0.04)	−0.01 (0.03)	−0.05 (0.04)	−0.12 (0.04)	0.03 (0.04)	0.07 (0.04)
BF			−0.08	0.41 (0.06)	0.002 (0.04)	−0.05 (0.04)	−0.01 (0.05)	−0.01 (0.04)	0.00 (0.04)	−0.01 (0.05)	−0.01 (0.05)	−0.03 (0.05)	0.07 (0.06)
REA				0.13	−0.08 (0.05)	0.10 (0.05)	0.11 (0.05)	0.12 (0.05)	0.00 (0.04)	−0.09 (0.05)	0.04 (0.05)	−0.01 (0.05)	0.00 (0.06)
KPH					−0.01 (0.04)	−0.04 (0.04)	−0.09 (0.05)	−0.08 (0.05)	0.00 (0.05)	−0.01 (0.05)	−0.04 (0.05)	−0.01 (0.05)	−0.01 (0.06)
CED						0.00 (0.03)	0.00 (0.03)	0.00 (0.03)	0.02 (0.02)	0.20 (0.03)	0.00 (0.02)	0.17 (0.03)	0.03 (0.03)
BW							0.60 (0.01)	0.53 (0.01)	0.00 (0.02)	0.00 (0.02)	0.27 (0.03)	0.03 (0.03)	−0.05 (0.03)
WW								0.58 (0.01)	−0.17 (0.02)	−0.18 (0.02)	0.67 (0.01)	−0.02 (0.02)	−0.01 (0.03)
YW									−0.03 (0.02)	−0.03 (0.03)	0.68 (0.01)	0.01 (0.03)	−0.01 (0.03)
CEM										0.22 (0.02)	0.06 (0.02)	0.00 (0.03)	−0.04 (0.03)
Milk											0.67 (0.02)	0.11 (0.03)	0.03 (0.04)
MWW												0.08 (0.03)	0.02 (0.03)
Stay													0.03 (0.04)

^1^ HCW = hot carcass weight; MARB = marbling; BF = 12th rib fat; KPH = internal fat; CED = calving ease direct; BW = birth weight; WW = weaning weight; YW = yearling weight; CEM = calving ease maternal; MWW = maternal weaning weight; Stay = stayability; Doc = docility maternal traits in this population.

## Data Availability

Not applicable.

## Supplementary Materials

The following are available online at https://www.mdpi.com/2076-2615/11/2/471/s1, Table S1: Significant hot carcass weight (HCW) markers that are within 100,000 base pairs of identified significant markers and previously reported QTL and genes for those locations. Table S2: Significant average hot carcass weight (HCW) markers that are within 100,000 base pairs of identified significant markers and previously reported QTL and genes for those locations. Table S3: Significant marbling (MARB) markers that are within 100,000 base pairs of identified significant markers and previously reported QTL and genes for those locations. Table S4: Significant average marbling (MARB) markers that are within 100,000 base pairs of identified significant markers and previously reported QTL and genes for those locations. Table S5: Significant 12th rib fat (BF) markers that are within 100,000 base pairs of identified significant markers and previously reported QTL and genes for those locations. Table S6: Significant average 12th rib fat (BF) markers that are within 100,000 base pairs of identified significant markers and previously reported QTL and genes for those locations. Table S7: Significant rib eye area (REA) markers that are within 100,000 base pairs of identified significant markers and previously reported QTL and genes for those locations. Table S8: Significant average rib eye area (REA) markers that are within 100,000 base pairs of identified significant markers and previously reported QTL and genes for those locations. Table S9: Significant internal fat (KPH) markers that are within 100,000 base pairs of identified significant markers and previously reported QTL and genes for those locations. Figure S1: Manhattan plot for hot carcass weight (HCW). Markers above –log10(*p*-value) of 5 × 10^−8^ are significant genome-wide association markers. Vertical clusters of markers are also of interest as they indicate potential QTL in those regions. Figure S2: Manhattan plot for average hot carcass weight (HCW). Markers above –log10(*p*-value) of 5 × 10^−8^ are significant genome-wide association markers. Vertical clusters of markers are also of interest as they indicate potential QTL in those regions. Figure S3: Manhattan plot for marbling (MARB). Markers above –log10(*p*-value) of 5 × 10^−8^ are significant genome-wide association markers. Vertical clusters of markers are also of interest as they indicate potential QTL in those regions. Figure S4: Manhattan plot for average marbling (MARB). Markers above –log10(*p*-value) of 5 × 10^−8^ are significant genome-wide association markers. Vertical clusters of markers are also of interest as they indicate potential QTL in those regions. Figure S5: Manhattan plot for 12th rib fat (BF). Markers above –log10(*p*-value) of 5 × 10^−8^ are significant genome-wide association markers. Vertical clusters of markers are also of interest as they indicate potential QTL in those regions. Figure S6: Manhattan plot for average 12th rib fat (BF). Markers above –log10(*p*-value) of 5 × 10^−8^ are significant genome-wide association markers. Vertical clusters of markers are also of interest as they indicate potential QTL in those regions. Figure S7: Manhattan plot for rib eye area (REA). Markers above –log10(*p*-value) of 5 × 10^−8^ are significant genome-wide association markers. Vertical clusters of markers are also of interest as they indicate potential QTL in those regions. Figure S8: Manhattan plot for average rib eye area (REA). Markers above –log10(*p*-value) of 5 × 10^−8^ are significant genome-wide association markers. Vertical clusters of markers are also of interest as they indicate potential QTL in those regions.
