# Supplementary material for: Identification of Genomic Regions for Carcass Quality Traits within the American Simmental Association Carcass Merit Program
Source: Animals (Basel). 2021 Feb 10;11(2):471. doi: 10.3390/ani11020471 (PMC7916785; doi:10.3390/ani11020471)
Supplement: Supplementary file 1 [file animals-11-00471-s001.pdf]

Supplementary Table S1. Significant hot carcass weight (HCW) markers that are within 100,000 base pairs of identified significant markers ($p < 0.001$) and previously reported QTL and genes for those locations.

| **Chromosome** | **Position (base pairs)** | **Positional Candidate Gene** | **Previously Identified QTL/Genes** | **Breed** | **Reference** |
|---|---|---|---|---|---|
| 1 | 34,450,639-34,492,226 | | | | |
| | 156,254,566-156,294,225 | | | | |
| 2 | 21,389,327-21,448,855 | | QTL 20354 | Angus, Charolais, University of Alberta hybrid bulls | [11] |
| | 37,729,012-37,813,654 | Bos taurus PKP4 | | | |
| | 133,258,339-133,349,015 | Bos taurus CAPZB | | | |
| 6 | 42,057,261-42,120,804 | | | | |
| | 89,104,201-89,133,819 | Bos taurus MTHFD2L | | | |
| 7 | 6,134,663-6,206,051 | Bos taurus F2RL3 | | | |
| | 20,423,138-20,500,709 | End of Bos taurus DOHH, Bos taurus SMIM24, Bos taurus LOC618071, Beginning of Bos taurus NFIC | | | |
| | 26,309,154-26,360,833 | End of Bos taurus CTXN3 | | | |
| | 83,621,039-83,648,346 | | | | |
| 9 | 56,406,855-56,490,122 | | | | |
| 10 | 12,659,662-12,707,435 | | | | |
| | 60,168,183-60,267,486 | | QTL 10876 | Commercial Angus | [12] |

Supplementary Table S1. Continued.

| Chromosome | Position (base pairs) | Positional Candidate Gene | Previously Identified QTL/Genes | Breed | Reference |
|---|---|---|---|---|---|
| 10 | 91,330,836-91,357,108 | | | | |
| 11 | 252,264-317,337 | | | | |
| 14 | 18,296,407-18,331,919 | | QTL 1733 | Waygu | [13] |
| 15 | 36,755,580-36,817,688 | | | | |
| | 61,893,000-61,927,861 | | | | |
| 16 | 2,813,940-2,841,879 | | QTL 11009 | Commercial Angus | [12] |
| 17 | 41,013,290-41,089,654 | | | | |
| | 41,089,654-41,178,453 | | | | |
| 19 | 57,098,859-57,128,225 | | | | |
| | 57,128,225-57,149,037 | | | | |
| 20 | 25,255,282-25,296,510 | | QTL 11107 | Commercial Angus | [12] |
| | 44,892,168-44,916,116 | | | | |
| | 58,292,591-58,329,179 | Beginning of Bos taurus ANKH | | | |
| | 64,169,690-64,185,456 | | | | |
| 22 | 56,603,472-56,675,252 | Bos taurus TSEN2, Beginning of Bos taurus PPARG-TSEN2 | | | |
| 25 | 40,022,986-40,060,928 | | | | |

Supplementary Table S1. Continued.

| Chromosome | Position (base pairs) | Positional Candidate Gene | Previously Identified QTL/Genes | Breed | Reference |
|---|---|---|---|---|---|
| 27 | 40,951,386-41,049,981 | | | | |
| | 41,191,284-41,226,347 | | | | |
| 28 | 38,961,890-39,061,596 | | | | |

Supplementary Table S2. Significant average hot carcass weight (HCW) markers that are within 100,000 base pairs of identified significant markers ($p < 0.001$) and previously reported QTL and genes for those locations.

| Chromosome | Position (base pairs) | Positional Candidate Gene | Previously Identified QTL/Genes | Breed | Reference |
|---|---|---|---|---|---|
| 1 | 8,347,160-8,377,141 | | | | |
| | 14,853,644-14,909,272 | | | | |
| | 50,252,331-50,320,575 | Bos taurus CBLB | | | |
| | 118,694,108-118,777,869 | | | | |
| | 138,528,244-138,612,242 | Bos taurus NUDT16 | | | |
| | 156,254,566-156,294,225 | | | | |
| 2 | 59,644,564-59,690,093 | | QTL 10665 | Commercial Angus | [12] |
| | | | QTL 13187 **PRKAG3** | Aberdeen Angus sired steers | [14] |
| 3 | 65,445,867-65,467,544 | | QTL 10699 | Commercial Angus | [12] |
| | 88,923,355-88,950,716 | | | | |
| | 116,968,644-117,023,378 | End of Bos taurus MLPH, Bos taurus PRLH | | | |
| 4 | 93,398,396-93,462,288 | Beginning of Bos taurus NRF1 | | | |
| 5 | 5,923,591-6,001,881 | | | | |
| | 117,795,427-117,823,521 | | | | |
| 6 | 14,094,866-14,120,051 | | QTL 10425, 10426, 10428, 10429 | Japanese Black | [15] |

Supplementary Table S2. Continued.

| Chromosome | Position (base pairs) | Positional Candidate Gene | Previously Identified QTL/Genes | Breed | Reference |
|---|---|---|---|---|---|
| 6 | 20,350,438-20,430,846 | | QTL 1369 | Belgian Blue x MARC III, Piedmontese x Angus | [16] |
| | | | QTL 10425, 10426, 10428, 10429 | Japanese Black | [15] |
| | 20,430,846-20,475,948 | | QTL 1369 | Belgian Blue x MARC III, Piedmontese x Angus | [16] |
| | | | QTL 10425, 10426, 10428, 10429 | Japanese Black | [15] |
| | | | QTL 24619 | Angus, Brangus, Charolais, Gelbvieh, Hereford, Limousin, Red Angus, Shorthorn, Maine, Simmental | [17] |
| 7 | 6,097,996-6,166,179 | | | | |
| | 16,588,162-16,611,621 | End of Bos taurus CD209, Beginning of Bos taurus EVI5L | QTL 10784 | Commercial Angus | [12] |
| | 62,508,803-62,599,314 | Bos taurus SLC36A2 | QTL 10809 | Commercial Angus | [12] |
| | 65,497,498-65,520,210 | | | | |
| | 106,398,519-106,422,549 | | | | |
| 8 | 64,438,267-64,476,783 | | | | |

Supplementary Table S2. Continued.

| Chromosome | Position (base pairs) | Positional Candidate Gene | Previously Identified QTL/Genes | Breed | Reference |
|---|---|---|---|---|---|
| 8 | 88,181,529-88,237,379 | | | | |
| 9 | 26,228,944-26,262,054 | | | | |
| | 28,337,856-28,400,169 | End of Bos taurus CLVS2 | | | |
| | 31,043,707-31,116,835 | | QTL 37238, 37240, 37250, 37255, 37259, 37264, 37267, 37275 | Holstein | [18] |
| | 100,827,855-100,892,769 | | | | |
| | 102,007,389-102,027,971 | Bos taurus UNC93A | | | |
| 10 | 66,659,307-66,687,859 | | QTL 10881 | Commercial Angus | [12] |
| | 68,089,016-68,162,634 | End of Bos taurus KTN1 | QTL 151847 | Commercial Angus | [12] |
| | 73,855,126-73,884,120 | End of Bos taurus HIF1A | | | |
| | 77,206,128-77,234,231 | End of Bos taurus MAX | | | |
| | 88,562,925-88,630,762 | End of Bos taurus TMED8, Beginning of Bos taurus VIPAS39 | | | |
| 11 | 54,855,548-54,887,301 | Bos taurus CTNNA2 | | | |
| 12 | 30,967,371-30,992,759 | Beginning of Bos taurus SLC7A1 | QTL 37280, 37281 – 37284 | Holstein | [18] |

Supplementary Table S2. Continued.

| Chromosome | Position (base pairs) | Positional Candidate Gene | Previously Identified QTL/Genes | Breed | Reference |
|---|---|---|---|---|---|
| 12 | 51,403,603-51,461,607 | Bos taurus MIR2285CC | | | |
| | 57,769,768-57,840,455 | | | | |
| | 90,483,656-90,536,166 | | | | |
| 13 | 8,347,568-8,386,291 | Bos taurus MACROD2 | QTL 10938 | Commercial Angus | [12] |
| | 10,368,225-10,400,443 | | QTL 10938 | Commercial Angus | [12] |
| | 23,478,182-23,529,663 | End of Bos taurus SPAG6 | | | |
| | 53,167,293-53,266,833 | Bos taurus SIRPA | | | |
| | 75,192,838-75,240,419 | | | | |
| 14 | 18,811,289-18,832,428 | | QTL 1733 | Waygu | [13] |
| | 58,589,050-58,687,826 | | | | |
| 15 | 71,342,153-71,375,213 | | | | |
| | 73,739,991-73,762,207 | | | | |
| | 81,540,854-81,595,546 | | | | |
| 16 | 69,167,269-69,202,739 | Bos taurus PTPN14 | | | |
| 17 | 4,306,870-4,347,226 | Beginning of Bos taurus TRIM2 | QTL 11040 | Commercial Angus | [12] |
| | 41,013,290-41,089,654 | | | | |

Supplementary Table S2. Continued.

| Chromosome | Position (base pairs) | Positional Candidate Gene | Previously Identified QTL/Genes | Breed | Reference |
|---|---|---|---|---|---|
| 17 | 41,089,654-41,178,453 | | | | |
| | 41,178,453-41,272,672 | | | | |
| | 73,206,420-73,257,794 | | | | |
| 18 | 9,891,406-9,948,949 | Bos taurus CDH13 | QTL 1336 | Sires: Hereford, Angus, Shorthorn, Charolais, Gelbvieh, Pinzgauer, Galloway, Longhorn, Nellore, Piedmontese, Saler<br>Dam: Hereford, Angus | [19] |
| | 22,574,534-22,610,574 | | | | |
| | 40,502,223-40,587,048 | Beginning of Bos taurus URI1 | | | |
| | 40,690,801-40,738,568 | | | | |
| 19 | 8,622,586-8,648,705 | | | | |
| | 58,551,913-58,591,027 | | | | |
| | 60,165,029-60,247,211 | | | | |
| 20 | 23,052,519-23,085,736 | Bos taurus ANKRD55 | QTL 11107 | Commercial Angus | [12] |
| | 58,240,835-58,264,762 | | | | |
| | 58,264,762-58,362,004 | Beginning of Bos taurus ANKH | | | |

Supplementary Table S2. Continued.

| Chromosome | Position (base pairs) | Positional Candidate Gene | Previously Identified QTL/Genes | Breed | Reference |
|---|---|---|---|---|---|
| 21 | 42,051,380-42,089,249 | Beginning of Bos taurus NUBPL | | | |
| 23 | 7,240,128-7,297,256 | Bos taurus BOLA-DMB, Bos taurus BOLA-DMA, Bos taurus BRD2 | QTL 11168 | Commercial Angus | [12] |
| | 28,792,687-28,819,118 | Bos taurus TRIM15, Beginning of Bos taurus TRIM10 | | | |
| | 35,606,289-35,642,738 | | | | |
| | 36,112,949-36,139,914 | | | | |
| 24 | 46,606,824-46,688,139 | | | | |
| 25 | 21,808,238-21,843,463 | Bos taurus PRKCB | QTL 11210 | Commercial Angus | [12] |
| 26 | 6,620,890-6,692,455 | | | | |
| 27 | 31,216,225-31,261,689 | Bos taurus UNC5D | | | |
| | 36,959,262-37,004,165 | | | | |
| | 39,916,848-39,994,067 | | | | |

Supplementary Table S2. Continued.

| Chromosome | Position (base pairs) | Positional Candidate Gene | Previously Identified QTL/Genes | Breed | Reference |
|---|---|---|---|---|---|
| 29 | 24,228,574-24,259,532 | | QTL 1316 **PRNP** | Brahman x Angus cross | [20] |
| | | | QTL 1344 | Sires: Hereford, Angus, Shorthorn, Charolais, Gelbvieh, Pinzgauer, Galloway, Longhorn, Nellore, Piedmontese, Saler<br>Dam: Hereford, Angus | [19] |
| | | | QTL 11292 | Commercial Angus | [12] |

Supplementary Table S3. Significant marbling (MARB) markers that are within 100,000 base pairs of identified significant markers and previously ($p < 0.001$) reported QTL and genes for those locations.

| Chromosome | Position (base pairs) | Positional Candidate Gene | Previously Identified QTL/Genes | Breed | Reference |
|---|---|---|---|---|---|
| 3 | 8,196,046-8,250,205 | End of Bos taurus SDHC, Bos taurus MPZ, Beginning of Bos taurus PCP4L1 | QTL 10060-10066 **RORC** | Angus, Shorthorn, other taurine | [21] |
| | | | QTL 11592 **RORC** | Angus, Hereford, Brahman | [22] |
| | 17,195,924-17,280,333 | Bos taurus LOR, Bos taurus LELP1 | | | |
| | 77,666,001-77,734,314 | | QTL 10700 | Commercial Angus | [12] |
| | 105,173,273-105,209,697 | End of Bos taurus SCMH1, Beginning of Bos taurus CTPS1 | | | |
| | 119,550,145-119,584,154 | | | | |
| | 121,251,149-121,310,309 | | | | |
| | 121,310,309-121,374,825 | | | | |
| 4 | 100,436,744-100,497,231 | | | | |
| 5 | 118,475,383-118,501,191 | End of Bos taurus FAM19A5 | | | |
| 6 | 14,094,866-14,120,051 | | | | |
| | 23,700,430-23,738,304 | Bos taurus PPP3CA | | | |
| | 35,577,199-35,611,267 | | QTL 10770 | Commercial Angus | [12] |
| | 66,509,207-66,572,393 | | | | |

Supplementary Table S3. Continued.

| Chromosome | Position (base pairs) | Positional Candidate Gene | Previously Identified QTL/Genes | Breed | Reference |
|---|---|---|---|---|---|
| 8 | 41,096,123-41,147,533 | | | | |
| | 76,530,816-76,582,220 | | | | |
| 9 | 10,765,698-10,838,261 | | QTL 4507 | Japanese Black | [23] |
| | | | QTL 10850 | Commercial Angus | [12] |
| | 34,943,071-35,002,290 | Bos taurus NT5DC1, Bos taurus COL10A1 | QTL 4906 | Sires: Hereford, Angus, Shorthorn, Charolais, Gelbvieh, Pinzgauer, Galloway, Longhorn, Nellore, Piedmontese, Saler Dam: Hereford, Angus | [19] |
| | 35,368,035-35,396,173 | | QTL 4906 | Sires: Hereford, Angus, Shorthorn, Charolais, Gelbvieh, Pinzgauer, Galloway, Longhorn, Nellore, Piedmontese, Saler Dam: Hereford, Angus | [19] |
| | 105,074,182-105,168,826 | | | | |
| 10 | 73,855,126-73,884,120 | Bos taurus HIF1A | | | |
| 11 | 78,755,260-78,831,137 | Bos taurus LAPTM4A | QTL 10909 | Commercial Angus | [12] |
| 13 | 43,009,513-43,054,682 | Bos taurus ASB13 | QTL 10947 | Commercial Angus | [12] |
| | 70,171,253-70,195,187 | Bos taurus CHD6 | | | |
| | 82,443,688-82,455,320 | | | | |

Supplementary Table S3. Continued.

| Chromosome | Position (base pairs) | Positional Candidate Gene | Previously Identified QTL/Genes | Breed | Reference |
|---|---|---|---|---|---|
| 14 | 49,385,228-49,450,613 | | | | |
| 15 | 55,185,032-55,254,553 | End of Bos taurus DGAT2, Beginning of Bos taurus UVRAG | | | |
| | 65,967,835-65,990,096 | Bos taurus PAMR1 | | | |
| | 76,930,018-76,960,713 | Bos taurus C15H11orf49 | | | |
| 16 | 2,246,201-2,329,194 | Bos taurus MDM4 | | | |
| 17 | 9,558,819-9,624,970 | Beginning of Bos taurus NR3C2 | QTL 1371 | Belgian Blue x MARC III, Piedmontese x Angus | [16] |
| | 13,386,146-13,455,790 | Beginning of Bos taurus HHIP | QTL 1371 | Belgian Blue x MARC III, Piedmontese x Angus | [16] |
| | 49,702,881-49,743,917 | | | | |
| 19 | 10,305,065-10,367,765 | Bos taurus YPEL2 | QTL 11077 | Commercial Angus | [12] |
| | 10,617,246-10,694,269 | End of Bos taurus CLTC, Bos taurus PTRH2, Beginning of Bos taurus VMP1 | QTL 11077 | Commercial Angus | [12] |
| | 19,637,405-19,718,709 | Beginning of Bos taurus NLK | QTL 11077 | Commercial Angus | [12] |
| | 19,718,709-19,789,422 | End of Bos taurus NLK | QTL 11077 | Commercial Angus | [12] |
| | 23,732,618-23,798,617 | | | | |
| 20 | 6,605,865-6,638,385 | | | | |

Supplementary Table S3. Continued.

| Chromosome | Position (base pairs) | Positional Candidate Gene | Previously Identified QTL/Genes | Breed | Reference |
|---|---|---|---|---|---|
| 20 | 18,631,431-18,719,808 | End of Bos taurus DEPDC1B | | | |
| | 18,719,808-18,743,927 | | | | |
| 21 | 59,853,238-59,875,525 | | | | |
| | 63,930,679-63,955,841 | | | | |
| 23 | 51,097,849-51,118,713 | | | | |
| 24 | 62,130,639-62,169,295 | | | | |
| 26 | 20,813,204-20,903,573 | Beginning of Bos taurus DNMBP | | | |
| | 26,234,033-26,296,160 | | QTL 11238 | Commercial Angus | [12] |
| 27 | 32,129,484-32,157,142 | Beginning of Bos taurus KCNU1 | QTL 1372 | Belgian Blue x MARC III, Piedmontese x Angus | [16] |
| | 36,959,262-37,004,165 | | | | |
| | 41,552,379-41,613,442 | | | | |
| 29 | 51,438,462-51,502,868 | | | | |

Supplementary Table S4. Significant average marbling (MARB) markers that are within 100,000 base pairs of identified significant markers ($p < 0.001$) and previously reported QTL and genes for those locations.

| Chromosome | Position (base pairs) | Positional Candidate Gene | Previously Identified QTL/Genes | Breed | Reference |
|---|---|---|---|---|---|
| 1 | 16,196,001-16,245,203 | | QTL 10636 | Commercial Angus | [12] |
| 2 | 21,290,918-21,389,327 | | QTL 20298, 20299, 20317, 20398, 20399, 20400 | Angus, Charolais, University of Alberta hybrid bulls | [11] |
| | 21,389,327-21,448,855 | | QTL 20298, 20299, 20317, 20398, 20399, 20400 | Angus, Charolais, University of Alberta hybrid bulls | [11] |
| | 85,427,167-85,452,055 | Beginning of Bos taurus PGAP1 | | | |
| 3 | 17,195,924-17,280,333 | Bos taurus LOR, Bos taurus LELP1 | | | |
| | 116,781,408-116,801,749 | | | | |
| | 116,943,803-116,968,664 | | | | |
| | 116,968,664-116,990,141 | Beginning of Bos taurus MLPH | | | |
| | 116,990,141-117,023,378 | End of Bos taurus MLPH | | | |
| 4 | 96,282,870-96,308,638 | | QTL 24661 | Angus, Brangus, Charolais, Gelbvieh, Hereford, Limousin, Red Angus, Shorthorn, Maine, Simmental | [17] |
| 5 | 118,430,785-118,475,383 | Bos taurus FAM19A5 | | | |
| 6 | 2,217,430-2,296,875 | | QTL 10756 | Commercial Angus | [12] |
| | 20,350,438-20,430,846 | | | | |

Supplementary Table S4. Continued

| Chromosome | Position (base pairs) | Positional Candidate Gene | Previously Identified QTL/Genes | Breed | Reference |
|---|---|---|---|---|---|
| 6 | 20,430,846-20,475,948 | | QTL 10498 | Hanwoo | [24] |
| 7 | 5,352,670-5,400,415 | End of Bos taurus B3GNT3 | | | |
| 8 | 58,505,244-58,594,366 | | | | |
| | 58,986,469-59,085,873 | | | | |
| | 106,245,419-106,291,686 | | | | |
| | 108,652,993-108,732,464 | Bos taurus BRINP1 | | | |
| | 108,732,464-108,772,548 | Bos taurus BRINP1 | | | |
| | 108,772,548-108,819,340 | Bos taurus BRINP1 | | | |
| 9 | 36,556,225-36,645,234 | End of Bos taurus MARCKS | QTL 4906 | Sires: Hereford, Angus, Shorthorn, Charolais, Gelbvieh, Pinzgauer, Galloway, Longhorn, Nellore, Piedmontese, Saler<br>Dam: Hereford, Angus | [19] |
| 10 | 34,316,340-34,407,598 | | QTL 10874 | Commercial Angus | [12] |
| | 34,407,598-34,438,367 | | QTL 10874 | Commercial Angus | [12] |
| 11 | 6,724,678-6,758,495 | End of Bos taurus IL1R2 | | | |
| | 106,708,013-106,741,315 | Beginning of Bos taurus CACNA1B | | | |
| 12 | 85,428,753-85,453,952 | Beginning of Bos taurus ARHGEF7 | | | |

Supplementary Table S4. Continued.

| Chromosome | Position (base pairs) | Positional Candidate Gene | Previously Identified QTL/Genes | Breed | Reference |
|---|---|---|---|---|---|
| 13 | 17,146,206-17,232,510 | | QTL 10941 | Commercial Angus | [12] |
| | 43,319,313-43,367,995 | | QTL 10947 | Commercial Angus | [12] |
| | 67,810,247-67,888,763 | | | | |
| 14 | 18,263,091-18,331,919 | | QTL 1334 | Sires: Hereford, Angus, Shorthorn, Charolais, Gelbvieh, Pinzgauer, Galloway, Longhorn, Nellore, Piedmontese, Saler<br>Dam: Hereford, Angus | [19] |
| | 18,331,919-18,408,275 | | QTL 1334 | Sires: Hereford, Angus, Shorthorn, Charolais, Gelbvieh, Pinzgauer, Galloway, Longhorn, Nellore, Piedmontese, Saler<br>Dam: Hereford, Angus | [19] |
| | 19,441,969-19,531,288 | Bos taurus PRKDC | QTL 1334 | Sires: Hereford, Angus, Shorthorn, Charolais, Gelbvieh, Pinzgauer, Galloway, Longhorn, Nellore, Piedmontese, Saler<br>Dam: Hereford, Angus | [19] |
| | 58,342,794-58,408,381 | | | | |
| | 58,589,050-58,687,826 | | | | |
| | 81,197,368-81,269,892 | Bos taurus TAF2 | | | |
| | 81,269,892-81,309,300 | End of Bos taurus DSCC1, Beginning of Bos taurus DEPTOR | | | |

Supplementary Table S4. Continued.

| Chromosome | Position (base pairs) | Positional Candidate Gene | Previously Identified QTL/Genes | Breed | Reference |
|---|---|---|---|---|---|
| 14 | 84,505,345-84,594,318 | | | | |
| 15 | 35,333,265-35,364,770 | | QTL 10999 | Commercial Angus | [12] |
| | 54,939,673-54,995,188 | Beginning of Bos taurus MOGAT2 | | | |
| | 82,558,282-82,655,879 | | | | |
| | 82,655,879-82,689,485 | | | | |
| 16 | 13,656,858-13,733,378 | | QTL 1353 | Belgian Blue x MARC III, Piedmontese x Angus | [16] |
| | 36,744,747-36,768,083 | Beginning of Bos taurus ATP1B1 | | | |
| | 36,768,083-36,817,218 | End of Bos taurus ATP1B1, Beginning of Bos taurus NME7 | | | |
| 17 | 41,178,453-41,272,672 | | | | |
| 18 | 22,574,534-22,610,574 | | | | |
| 19 | 8,622,586-8,648,705 | | QTL 11077 | Commercial Angus | [12] |
| 20 | 15,454,163-15,528,205 | | | | |
| 21 | 9,741,507-9,784,549 | | | | |
| 22 | 56,603,472-56,675,252 | Bos taurus TSEN2, Beginning of Bos taurus PPARG-TSEN2 | | | |

Supplementary Table S4. Continued.

| Chromosome | Position (base pairs) | Positional Candidate Gene | Previously Identified QTL/Genes | Breed | Reference |
|---|---|---|---|---|---|
| 23 | 3,626,219-3,664,434 | | | | |
| 24 | 24,904,038-24,961,929 | | | | |
| 26 | 26,234,033-26,267,018 | | QTL 11238 | Commercial Angus | [12] |
| 27 | 1,271,954-1,312,576 | | | | |
| | 8,589,473-8,689,042 | End of Bos taurus NEIL3, Beginning of Bos taurus AGA | QTL 1342 | Sires: Hereford, Angus, Shorthorn, Charolais, Gelbvieh, Pinzgauer, Galloway, Longhorn, Nellore, Piedmontese, Saler<br>Dam: Hereford, Angus | [19] |
| | 20,791,916-20,863,121 | | QTL 1342 | Sires: Hereford, Angus, Shorthorn, Charolais, Gelbvieh, Pinzgauer, Galloway, Longhorn, Nellore, Piedmontese, Saler<br>Dam: Hereford, Angus | [19] |
| | 30,479,059-30,510,177 | | QTL 11258<br><br>QTL 1372 | Commercial Angus<br><br>Belgian Blue x MARC III, Piedmontese x Angus | [12]<br><br>[16] |
| | 31,216,225-31,261,689 | Bos taurus UNC5D | QTL 1372 | Belgian Blue x MARC III, Piedmontese x Angus | [16] |
| | 32,129,484-32,157,142 | Beginning of Bos taurus KCNU1 | QTL 1372 | Belgian Blue x MARC III, Piedmontese x Angus | [16] |
| | 36,935,085-36,959,262 | | | | |
| | 41,613,442-41,649,895 | | | | |

Supplementary Table S4. Continued.

| Chromosome | Position (base pairs) | Positional Candidate Gene | Previously Identified QTL/Genes | Breed | Reference |
|---|---|---|---|---|---|
| 28 | 8,080,087-8,105,051 | Beginning of Bos taurus TBCE | QTL 11270 | Commercial Angus | [12] |
| | 16,097,749-16,097,772 | | QTL 11270 | Commercial Angus | [12] |

Supplementary Table S5. Significant 12th rib fat (BF) markers that are within 100,000 base pairs of identified significant markers ($p < 0.001$) and previously reported QTL and genes for those locations.

| Chromosome | Position (base pairs) | Positional Candidate Gene | Previously Identified QTL/Genes | Breed | Reference |
|---|---|---|---|---|---|
| 1 | 34,450,639-34,492,226 | | QTL 4854 | Waygu x Limousin | [25] |
| | 47,123,160-47,177,862 | | | | |
| | 76,505,437-76,534,601 | | | | |
| | 81,160,609-81,239,683 | End of Bos taurus FETUB ETV5 | QTL 10649 | Commercial Angus | [12] |
| | 127,998,001-128,031,876 | Beginning of Bos taurus SLC25A36 | | | |
| | 128,864,268-128,938,068 | Bos taurus CLSTN2 | | | |
| | 131,642,361-131,684,053 | | | | |
| 3 | 81,428,130-81,472,159 | | QTL 10701 | Commercial Angus | [12] |
| 4 | 5,639,667-5,712,778 | | | | |
| | 62,549,908-62,606,492 | | | | |
| | 74,076,693-74,148,361 | | | | |
| | 80,350,223-80,407,501 | Bos taurus SUGCT | | | |
| 5 | 12,174,285-12,213,478 | | | | |
| | 118,307,910-118,392,147 | Bos taurus FAM19A5 | | | |

Supplementary Table S5. Continued.

| Chromosome | Position (base pairs) | Positional Candidate Gene | Previously Identified QTL/Genes | Breed | Reference |
|---|---|---|---|---|---|
| 7 | 38,997,984-39,060,855 | Bos taurus DOK3, Bos taurus DDX41, Bos taurus FAM193B | | | |
| 8 | 91,086,691-91,125,290 | Bos taurus MRPL50, Beginning of Bos taurus ZNF189 | | | |
| | 104,829,510-104,862,806 | | | | |
| 9 | 92,242,453-92,250,839 | | | | |
| | 98,306,160-98,349,764 | | | | |
| 10 | 102,849,006-102,935,702 | End of Bos taurus CASC4, Beginning of Bos taurus CTDSPL2 | | | |
| 11 | 91,995,272-92,059,274 | | | | |
| | 95,501,827-95,516,698 | Beginning of Bos taurus NR5A1 | | | |
| | 98,407,974-98,482,863 | Bos taurus TTC16, Bos taurus TOR2A, Bos taurus SH2D3C, Bos taurus CDK9 | | | |
| 12 | 60,040,871-60,134,810 | | | | |
| 13 | 48,594,834-48,622,655 | | | | |
| | 65,761,897-65,855,988 | End of Bos taurus NDRG3, Bos taurus DSN1 | | | |
| 14 | 27,271,835-27,321,716 | | QTL 10965 | Commercial Angus | [12] |
| | 27,669,598-27,751,888 | | QTL 10965 | Commercial Angus | [12] |

Supplementary Table S5. Continued.

| Chromosome | Position (base pairs) | Positional Candidate Gene | Previously Identified QTL/Genes | Breed | Reference |
|---|---|---|---|---|---|
| 14 | 48,184,967-48,256,343 | | | | |
| | 70,250,637-70,314,452 | | | | |
| | 75,517,287-75,571,250 | | | | |
| 15 | 34,294,990-34,342,385 | | | | |
| 16 | 66,609,042-66,676,299 | | | | |
| 17 | 5,590,969-5,663,467 | | | | |
| | 7,756,498-7,794,593 | | | | |
| 19 | 16,578,349-16,598,801 | Bos taurus ASIC2 | | | |
| 20 | 28,850,177-28,887,439 | | QTL 15736 | Angus x Brahman | [26] |
| | | | QTL 20540, 20542 | Brangus | [27] |
| 21 | 27,927,781-27,956,825 | | QTL 11125 | Commercial Angus | [12] |
| | 27,956,825-28,055,227 | Beginning of Bos taurus APBA2 | QTL 11125 | Commercial Angus | [12] |
| 22 | 60,105,535-60,130,492 | Bos taurus EEFSEC | | | |
| 23 | 41,901,234-41,965,164 | | | | |
| | 52,038,120-52,068,611 | | | | |
| | 52,068,611-52,128,894 | Beginning of Bos taurus EXOC2 | | | |

Supplementary Table S5. Continued.

| Chromosome | Position (base pairs) | Positional Candidate Gene | Previously Identified QTL/Genes | Breed | Reference |
|---|---|---|---|---|---|
| 24 | 61,885,106-61,931,908 | Beginning of Bos taurus LOC511106 | | | |
| 27 | 32,541,258-32,561,963 | | | | |
| 28 | 6,499,231-6,547,497 | Beginning of Bos taurus KCNK1 | | | |
| | 21,014,194-21,066,992 | | QTL 20273 | Crosses of Angus, Charolais, University of Alberta hybrid bulls | [11] |

Supplementary Table S6. Significant average 12th rib fat (BF) markers that are within 100,000 base pairs of identified significant markers ($p < 0.001$) and previously reported QTL and genes for those locations.

| **Chromosome** | **Position (base pairs)** | **Positional Candidate Gene** | **Previously Identified QTL/Genes** | **Breed** | **Reference** |
|---|---|---|---|---|---|
| 1 | 1,288,510-1,359,951 | | QTL 1317 **IFNAR1** | Brahman x Angus cross | [20] |
| | 14,853,644-14,909,272 | | | | |
| | 50,252,331-50,320,575 | Beginning of Bos taurus CBLB | | | |
| | 100,513,127-100,605,192 | | QTL 10653 | Commercial Angus | [12] |
| 2 | 16,157,387-16,238,330 | | QTL 1322 | Sires: Hereford, Angus, Shorthorn, Charolais, Gelbvieh, Pinzgauer, Galloway, Longhorn, Nellore, Piedmontese, Saler Dam: Hereford, Angus | [19] |
| | 111,103,057-111,181,420 | End of Bos taurus ACSL3 | | | |
| 3 | 10,956,984-11,040,167 | Beginning of Bos taurus SPTA1 | | | |
| | 17,241,980-17,280,333 | Bos taurus LELP1 | QTL 1325 | Sires: Hereford, Angus, Shorthorn, Charolais, Gelbvieh, Pinzgauer, Galloway, Longhorn, Nellore, Piedmontese, Saler Dam: Hereford, Angus | [19] |
| | 82,037,605-82,113,195 | End of Bos taurus EFCAB7, Beginning of Bos taurus ITGB3BP | QLT 10701 | Commercial Angus | [12] |
| 3 | 89,579,376-89,675,602 | Beginning of Bos taurus PLPP3 | | | |
| | 112,922,761-112,955,526 | Beginning of Bos taurus INPP5D | | | |

Supplementary Table S6. Continued.

| Chromosome | Position (base pairs) | Positional Candidate Gene | Previously Identified QTL/Genes | Breed | Reference |
|---|---|---|---|---|---|
| 3 | 115,730,569-115,790,125 | End of Bos taurus ASB18, Beginning of Bos taurus IQCA1 | | | |
| | 116,968,644-117,023,378 | End of Bos taurus MLPH, Bos taurus PRLH | | | |
| 4 | 21,937,293-21,973,829 | | QTL 20280, 20285, 20368, 20446 | Crosses of Angus, Charolais, University of Alberta hybrid bulls | [11] |
| | 96,258,114-96,282,870 | | | | |
| 5 | 6,976,839-7,053,234 | | QTL 20001 **MYF5** | Jiaxian Red, Qinchuan, Luxi, Nanyang, Xianan | [28] |
| | 97,884,594-97,972,722 | Beginning of Bos taurus ETV6 | | | |
| 6 | 20,276,795-20,297,397 | | QTL 24647 | Angus, Brangus, Charolais, Gelbvieh, Hereford, Limousin, Red Angus, Shorthorn, Maine, Simmental | [17] |
| | 44,305,092-44,337,791 | | | | |
| | 107,016,833-107,095,193 | | | | |
| 7 | 12,833,745-12,889,689 | End of Bos taurus WDR83OS, Bos taurus MAN2B1 | | | |
| | 66,156,674-66,212,415 | | | | |
| | 66,212,415-66,254,499 | | | | |
| 8 | 37,081,128-37,158,190 | Bos taurus DMAC1 | | | |

Supplementary Table S6. Continued.

| Chromosome | Position (base pairs) | Positional Candidate Gene | Previously Identified QTL/Genes | Breed | Reference |
|---|---|---|---|---|---|
| 8 | 81,573,118-81,612,097 | Bos taurus C8H9orf3 | | | |
| | 112,323,763-112,345,659 | | | | |
| 9 | 31,043,707-31,067,189 | | | | |
| | 74,223,999-74,293,236 | Bos taurus MTFR2, Bos taurus BCLAF1 | | | |
| | 81,166,662-81,216,232 | | | | |
| 10 | 66,659,307-66,687,859 | | | | |
| | 101,953,883-101,984,056 | | | | |
| | 102,887,596-102,935,702 | Bos taurus CTDSPL2 | | | |
| 11 | 103,799,585-103,830,982 | Beginning of Bos taurus CARD9 | | | |
| 12 | 48,909,344-48,984,802 | | | | |
| | 48,984,802-49,006,756 | | | | |
| | 49,006,756-49,057,460 | | | | |
| 12 | 49,057,460-49,095,991 | | | | |
| | 82,765,192-82,812,721 | Bos taurus MIR2301, Beginning of Bos taurus FAM155A | | | |
| 14 | 33,580,690-33,604,665 | | QTL 10971 | Commercial Angus | [12] |

Supplementary Table S6. Continued.

| Chromosome | Position (base pairs) | Positional Candidate Gene | Previously Identified QTL/Genes | Breed | Reference |
|---|---|---|---|---|---|
| 14 | 58,589,050-58,687,826 | | | | |
| 15 | 41,707,747-41,757,348 | Bos taurus GALNT18 | | | |
| | 80,003,887-80,097,824 | | | | |
| 16 | 1,199,397-1,296,954 | Bos taurus PRELP, Bos taurus OPTC | | | |
| | 31,382,509-31,462,716 | Bos taurus SMYD3 | | | |
| 17 | 4,868,261-4,926,550 | End of Bos taurus ARFIP1 | | | |
| | 34,409,623-34,429,947 | | | | |
| | 41,178,453-41,272,672 | | QTL 11052 | Commercial Angus | [12] |
| | 49,702,881-49,791,728 | | | | |
| | 70,699,234-70,776,835 | | | | |
| | 72,710,280-72,790,867 | End of Bos taurus UFD1, Bos taurus CDC45, Bos taurus CLDN5, End of Bos taurus SEPT5 | | | |
| 18 | 3,146,088-3,177,824 | | QTL 11058 | Commercial Angus | [12] |
| 19 | 5,057,128-5,083,994 | End of Bos taurus TOM1L1, Beginning of Bos taurus COX11 | | | |
| | 22,749,859-22,771,407 | Beginning of Bos taurus PRPF8 | | | |

Supplementary Table S6. Continued.

| Chromosome | Position (base pairs) | Positional Candidate Gene | Previously Identified QTL/Genes | Breed | Reference |
|---|---|---|---|---|---|
| 19 | 22,771,407-22,864,442 | End of Bos taurus PRPF8, Bos taurus TLCD2, Bos taurus MIR22, Bos taurus MIR3600, Bos taurus SERPINF2, Bos taurus SERPINF1, Beginning of Bos taurus SMYD4 | | | |
| | 33,862,979-33,895,220 | Bos taurus ALDH3A1 | | | |
| | 58,551,913-58,591,027 | | | | |
| 20 | 18,631,431-18,719,808 | End of Bos taurus DEPDC1B | | | |
| | 18,719,808-18,743,927 | | | | |
| | 22,093,001-22,165,165 | | QTL 20540, 20542 | Brangus | [27] |
| | 57,373,160-57,403,850 | | | | |
| 21 | 58,994,572-58,996,585 | | | | |
| 22 | 499,356-574,301 | Beginning of Bos taurus VOPP1 | | | |
| | 56,765,689-56,833,606 | Beginning of Bos taurus PPARG-TSEN2, Beginning of Bos taurus PPARG, Bos taurus MIR2373 | | | |
| 22 | 56,833,606-56,916,823 | | | | |
| | 58,397,411-58,429,229 | Beginning of Bos taurus HDAC11 | | | |

Supplementary Table S6. Continued.

| Chromosome | Position (base pairs) | Positional Candidate Gene | Previously Identified QTL/Genes | Breed | Reference |
|---|---|---|---|---|---|
| 23 | 45,048,666-45,106,522 | Beginning of Bos taurus NEDD9 | | | |
| 24 | 1,529,327-1,582,182 | | | | |
| | 62,104,076-62,130,639 | End of Bos taurus SERPINB10 | | | |
| 25 | 17,860,710-17,891,876 | | | | |
| 26 | 22,557,427-22,628,269 | | | | |
| 27 | 8,589,473-8,649,727 | End of Bos taurus NEIL3 | | | |
| | 8,649,727-8,689,042 | Beginning of Bos taurus AGA | | | |
| | 11,657,439-11,715,052 | | | | |
| 28 | 6,499,231-6,547,497 | End of Bos taurus KCNK1 | | | |
| | 7,001,292-7,068,698 | Bos taurus SCL35F3 | | | |
| | 7,068,698-7,138,132 | Bos taurus SCL35F3 | | | |

Supplementary Table S7. Significant rib eye area (REA) markers that are within 100,000 base pairs of identified significant markers ($p < 0.001$) and previously reported QTL and genes for those locations.

| Chromosome | Position (base pairs) | Positional Candidate Gene | Previously Identified QTL/Genes | Breed | Reference |
|---|---|---|---|---|---|
| 1 | 122,789,893-122,814,776 | | | | |
| | 143,741,734-143,831,554 | Bos taurus PDXK, Bos taurus CSTB | | | |
| | 145,643,805-145,682,206 | Beginning of Bos taurus COL6A1 | | | |
| | 145,682,206-145,710,047 | End of Bos taurus COL6A1 | | | |
| 3 | 82,187,624-82,250,730 | End of Bos taurus ALG6 | | | |
| 7 | 16,588,162-16,611,621 | End of Bos taurus CD209, Bos taurus EVI5L | | | |
| | 60,436,313-60,475,602 | | | | |
| | 78,762,342-78,822,792 | | | | |
| | 84,925,417-84,991,860 | | | | |
| 8 | 19,811,075-19,863,242 | | | | |
| | 37,136,374-37,158,190 | | | | |
| 9 | 99,334,002-99,354,827 | | | | |
| 10 | 32,409,981-32,433,588 | Bos taurus C10H15orf41 | | | |
| | 88,563,925-88,605,589 | End of Bos taurus TMED8 | | | |
| 11 | 20,202,555-20,271,885 | | | | |
| | 22,688,045-22,716,948 | Bos taurus SLC8A1 | | | |

Supplementary Table S7. Continued.

| Chromosome | Position (base pairs) | Positional Candidate Gene | Previously Identified QTL/Genes | Breed | Reference |
|---|---|---|---|---|---|
| 11 | 75,586,338-75,662,200 | | | | |
| 12 | 4,324,502-4,397,035 | | | | |
| | 86,938,949-86,961,976 | Bos taurus RASA3 | | | |
| 13 | 79,914,560-79,991,041 | | | | |
| 14 | 20,323,857-20,347,849 | | | | |
| | 27,575,294-27,669,598 | | | | |
| 15 | 34,763,290-34,852,656 | End of Bos taurus KCNC1, Bos taurus MYOD1 | | | |
| | 37,237,274-37,309,941 | | | | |
| | 55,206,099-55,254,553 | Beginning of Bos taurus UVRAG | | | |
| | 56,571,496-56,663,853 | | | | |
| 16 | 38,807,074-38,830,470 | Beginning of Bos taurus FMO1 | | | |
| 17 | 13,941,499-13,984,972 | | | | |
| | 41,089,654-41,178,453 | | | | |
| 20 | 25,255,282-25,296,510 | | | | |
| | 43,579,015-43,649,910 | | | | |
| 23 | 46,334,617-46,401,353 | | | | |

Supplementary Table S7. Continued.

| Chromosome | Position (base pairs) | Positional Candidate Gene | Previously Identified QTL/Genes | Breed | Reference |
|---|---|---|---|---|---|
| 23 | 52,038,120-52,128,894 | Beginning of Bos taurus EXOC2 | | | |
| 25 | 28,974,015-29,065,778 | Bos taurus CALN1 | | | |
| 26 | 37,733,926-37,797,893 | | | | |

Supplementary Table S8. Significant average rib eye area (REA) markers that are within 100,000 base pairs of identified significant markers ($p < 0.001$) and previously reported QTL and genes for those locations.

| Chromosome | Position (base pairs) | Positional Candidate Gene | Previously Identified QTL/Genes | Breed | Reference |
|---|---|---|---|---|---|
| 1 | 48,726,549-48,802,718 | | | | |
| | 114,085,424-114,180,799 | | | | |
| | 151,835,916-151,858,713 | | | | |
| 2 | 9,810,493-9,867,063 | Bos taurus ZC3H15 | | | |
| | 15,250,050-15,303,929 | | | | |
| | 18,164,650-18,233,729 | | | | |
| | 29,369,336-29,405,628 | | | | |
| | 59,644,564-59,690,093 | | | | |
| 3 | 62,458,422-62,509,283 | Bos taurus ADGRL2 | | | |
| 4 | 15,468,000-15,537,254 | | | | |
| | 28,350,426-28,413,603 | Bos taurus TWISTNB | | | |
| 5 | 109,134,816-109,167,093 | Bos taurus BID | | | |
| | 117,738,204-117,795,427 | | | | |
| | 119,949,553-120,016,258 | Beginning of Bos taurus ACR | | | |
| 7 | 2,323,494-2,346,546 | Beginning of Bos taurus ZNF879 | | | |
| | 3,488,080-3,570,752 | | | | |
| | 56,055,399-56,152,302 | | | | |

Supplementary Table S8. Continued.

| Chromosome | Position (base pairs) | Positional Candidate Gene | Previously Identified QTL/Genes | Breed | Reference |
|---|---|---|---|---|---|
| 7 | 78,762,342-78,822,792 | | | | |
| | 78,822,792-78,877,775 | | | | |
| | 80,229,987-80,307,024 | | | | |
| 8 | 81,612,097-81,638,162 | End of Bos taurus C8H9orf3, Bos taurus MIR2475, Bos taurus MIR23B, Bos taurus MIR276, Bos taurus MIR24-1, Beginning of Bos taurus FANCC | | | |
| 9 | 14,794,389-14,817,532 | | | | |
| | 98,566,722-98,648,213 | | | | |
| 10 | 61,648,704-61,769,231 | Beginning of Bos taurus FBN1 | | | |
| | 68,089,016-68,162,634 | End of Bos taurus KTN1 | | | |
| | 68,162,634-68,231,955 | | | | |
| | 72,023,342-72,060,561 | | | | |
| | 88,563,925-88,630,762 | End of Bos taurus TMED8, Beginning of Bos taurus VIPAS39 | | | |
| | 90,543,010-90,589,157 | Bos taurus NRXN3 | | | |
| 11 | 41,114,000-41,204,355 | | | | |
| | 54,855,548-54,887,301 | Bos taurus CTNNA2 | | | |
| | 55,229,674-55,264,686 | Bos taurus CTNNA2 | | | |

Supplementary Table S8. Continued.

| Chromosome | Position (base pairs) | Positional Candidate Gene | Previously Identified QTL/Genes | Breed | Reference |
|---|---|---|---|---|---|
| 11 | 71,811,673-71,875,673 | End of Bos taurus BABAM2, Beginning of Bos taurus RBKS | | | |
| 12 | 77,964,481-78,014,490 | Bos taurus ITGBL1 | | | |
| 13 | 2,342,625-2,372,577 | Bos taurus PLCB4 | | | |
| | 4,634,576-4,733,016 | | | | |
| | 37,675,851-37,725,131 | Bos taurus PCSK2 | | | |
| | 37,725,131-37,757,395 | End of Bos taurus PCSK2, Beginning of Bos taurus LOC515755 | | | |
| | 57,524,735-57,570,093 | End of Bos taurus GNAS | | | |
| | 59,963,672-60,002,265 | Beginning of Bos taurus PSMF1 | | | |
| 14 | 2,194,228-2,239,085 | | | | |
| | 18,811,289-18,832,428 | | | | |
| | 19,132,330-19,172,385 | | | | |
| 15 | 73,739,991-73,762,207 | | | | |
| 16 | 933,282-950,232 | Bos taurus ADORA1 | | | |
| | 61,668,331-61,716,582 | End of Bos taurus ACBD6 | | | |
| 17 | 49,702,881-49,791,728 | | | | |
| | 63,885,514-63,939,534 | Bos taurus ACACB | | | |
| 20 | 53,635,068-53,674,655 | Bos taurus CDH18 | | | |
| | 69,430,215-69,459,313 | | | | |
| 22 | 6,450,949-6,485,383 | | | | |

Supplementary Table S8. Continued.

| Chromosome | Position (base pairs) | Positional Candidate Gene | Previously Identified QTL/Genes | Breed | Reference |
|---|---|---|---|---|---|
| 22 | 51,214,861-51,245,935 | Beginning of Bos taurus CELSR3 | | | |
| | 51,452,218-51,484,825 | | | | |
| 23 | 32,682,177-32,757,561 | | | | |
| | 51,621,297-51,719,827 | | | | |
| | 52,068,611-52,091,670 | Beginning of Bos taurus EXOC2 | | | |
| 24 | 60,745,394-60,842,899 | | | | |
| 25 | 40,282,215-40,341,603 | | | | |
| 26 | 9,669,992-9,725,747 | | | | |
| 27 | 37,283,994-37,357,125 | End of Bos taurus SLC20A2 | | | |
| | 37,357,125-37,389,551 | Bos taurus SMIM19 | | | |
| | 37,389,551-37,430,965 | | | | |
| | 39,916,848-39,994,067 | | | | |
| 29 | 21,987,120-22,019,432 | | | | |

Supplementary Table S9. Significant internal fat (KPH) markers that are within 100,000 base pairs of identified significant markers ($p < 0.001$) and previously reported QTL and genes for those locations.

| Chromosome | Position (base pairs) | Positional Candidate Gene | Previously Identified QTL/Genes | Breed | Reference |
|---|---|---|---|---|---|
| 1 | 883,895-950,841 | | | | |
| | 10,404,023-10,468,415 | Bos taurus APP | | | |
| | 16,145,053-16,169,001 | | | | |
| | 118,694,108-118,714,300 | | | | |
| | 142,401,535-142,446,153 | | | | |
| 2 | 30,262,141-30,307,800 | Bos taurus SCN1A | | | |
| 4 | 53,316,893-53,400,639 | | | | |
| | 117,292,302-117,339,600 | | | | |
| 6 | 3,093,621-3,149,732 | | | | |
| | 27,158,687-27,183,822 | | QTL 12153 | Jersey x Limousin | [29] |
| | 30,782,962-30,832,561 | | QTL 12153 | Jersey x Limousin | [29] |
| | 41,343,408-41,443,081 | Bos taurus KCNIP4 | QTL 12153 | Jersey x Limousin | [29] |
| | 42,155,077-42,239,393 | | QTL 12153 | Jersey x Limousin | [29] |
| | 44,622,597-44,649,549 | | QTL 12153 | Jersey x Limousin | [29] |
| 6 | 66,509,207-66,572,393 | | | | |

Supplementary Table S9. Continued.

| Chromosome | Position (base pairs) | Positional Candidate Gene | Previously Identified QTL/Genes | Breed | Reference |
|---|---|---|---|---|---|
| 8 | 40,775,647-40,800,617 | | | | |
| | 51,330,787-51,369,892 | | | | |
| | 73,881,694-73,907,982 | | | | |
| | 101,044,054-101,135,756 | Bos taurus DNAJC25, Bos taurus GNG10 | | | |
| | 101,135,756-101,167,884 | | | | |
| 9 | 55,740,550-55,802,932 | | | | |
| 10 | 92,952,608-92,984,267 | | | | |
| 11 | 15,919,622-15,945,389 | End of Bos taurus LTBP1 | | | |
| 12 | 45,919,459-45,952,853 | | | | |
| 13 | 49,963,611-50,004,272 | | | | |
| | 62,881,877-62,909,025 | End of Bos taurus CDK5RAP1, Beginning of Bos taurus SNTA1 | | | |
| 14 | 59,112,331-59,139,878 | | | | |
| 16 | 37,479,436-37,505,165 | Beginning of Bos taurus SCYL3 | QTL 1354 | (Brahman x Angus) x Hereford, Angus, MARC III | [30] |
| 17 | 1,180,289-1,261,843 | | | | |
| | 64,189,856-64,225,341 | Beginning of Bos taurus CORO1C | | | |

Supplementary Table S9. Continued.

| Chromosome | Position (base pairs) | Positional Candidate Gene | Previously Identified QTL/Genes | Breed | Reference |
|---|---|---|---|---|---|
| 17 | 68,952,931-69,030,893 | Beginning of Bos taurus MTMR3 | | | |
| 20 | 26,330,033-26,397,183 | | QTL 12157 | Jersey x Limousin | [29] |
| 21 | 7,283,843-7,311,519 | Bos taurus LRRC28 | | | |
| | 57,819,236-57,848,290 | Bos taurus GON7, Bos taurus UBR7 | | | |
| 22 | 37,615,930-37,652,444 | End of Bos taurus THOC7 | | | |
| 24 | 56,487,933-56,564,480 | | | | |
| 25 | 40,022,986-40,060,928 | | | | |
| 26 | 6,051,502-6,092,833 | | | | |
| 27 | 30,025,162-30,089,811 | | | | |
| 29 | 41,778,946-41,854,768 | | | | |
| | 42,620,218-42,696,595 | Bos taurus CCDC88B, Bos taurus RPS6KA4 | | | |
| | 42,985,739-43,043,207 | End of Bos taurus PYGM, Bos taurus SF1, Bos taurus MAP4K2, Beginning of Bos taurus MEN1 | | | |

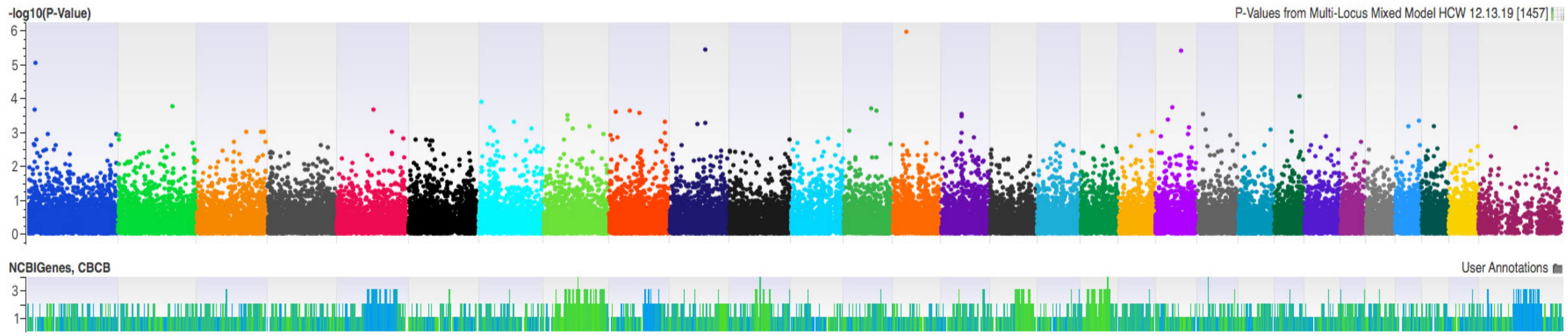


Supplementary Figure S1. Manhattan plot for hot carcass weight (HCW). Markers above –log10(*p*-value) of 5 x $10^{-8}$ are genome-wide association significant markers. Vertical clusters of markers are also of interest as they are indicating suggestive QTL in those regions.

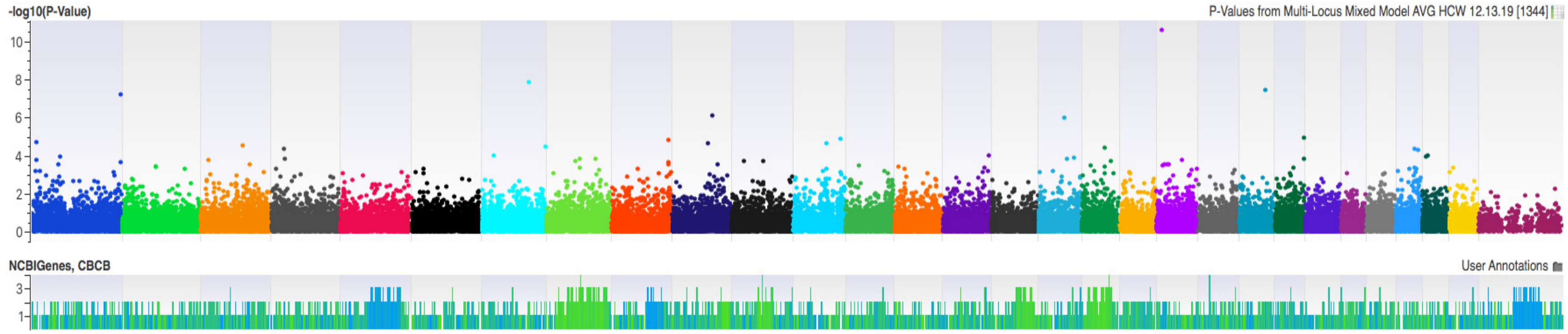


Supplementary Figure S2. Manhattan plot for average hot carcass weight (HCW). Markers above –log10(*p*-value) of 5 x $10^{-8}$ are genome-wide association significant markers. Vertical clusters of markers are also of interest as they are indicating suggestive QTL in those regions.

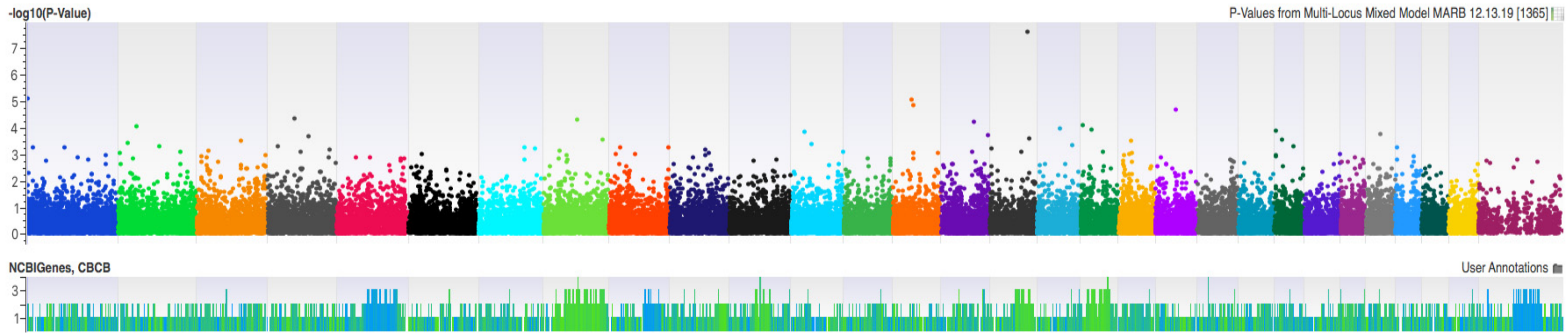


Supplementary Figure S3. Manhattan plot for marbling (MARB). Markers above –log10(*p*-value) of 5 x $10^{-8}$ are genome-wide association significant markers. Vertical clusters of markers are also of interest as they are indicating suggestive QTL in those regions.

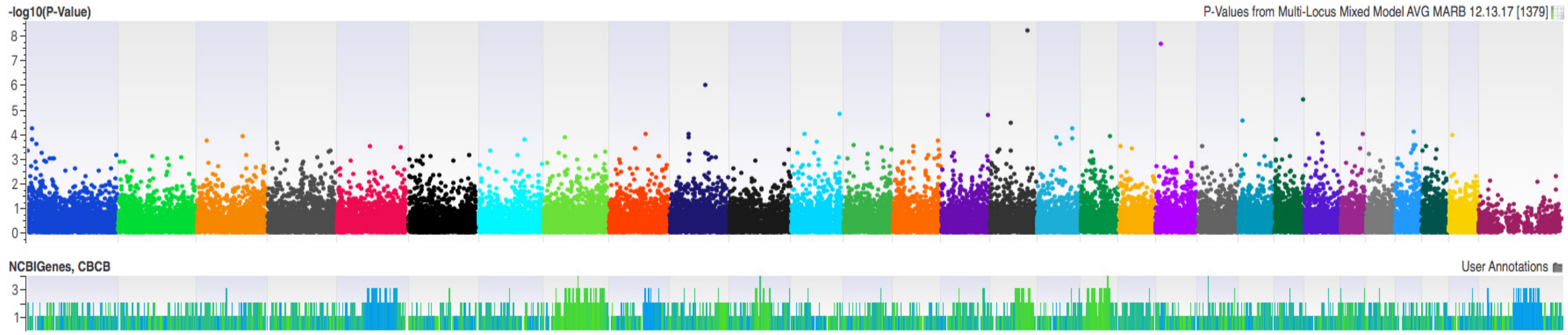


Supplementary Figure S4. Manhattan plot for average marbling (MARB). Markers above –log10(*p*-value) of 5 x $10^{-8}$ are genome-wide association significant markers. Vertical clusters of markers are also of interest as they are indicating suggestive QTL in those regions.

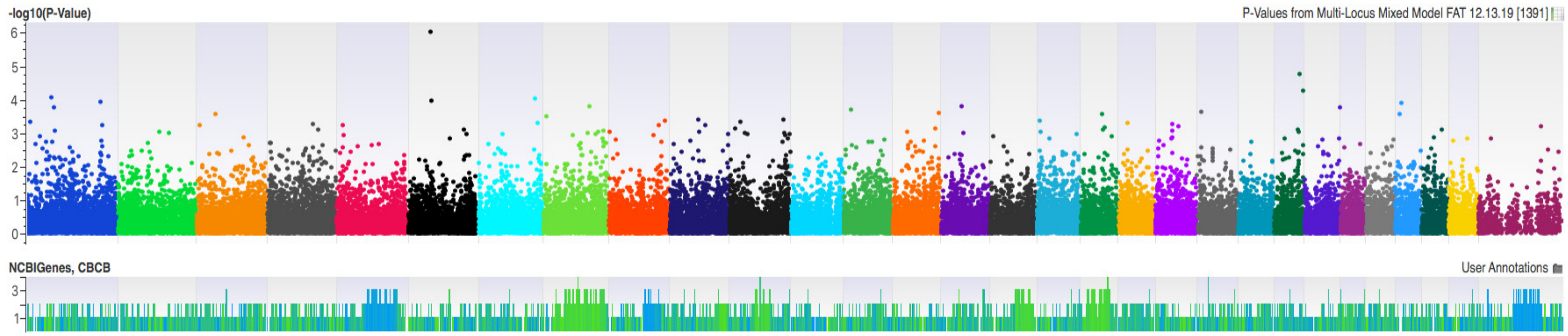


Supplementary Figure S5. Manhattan plot for 12th rib fat (BF). Markers above –log10(*p*-value) of 5 x $10^{-8}$ are genome-wide association significant markers. Vertical clusters of markers are also of interest as they are indicating suggestive QTL in those regions.

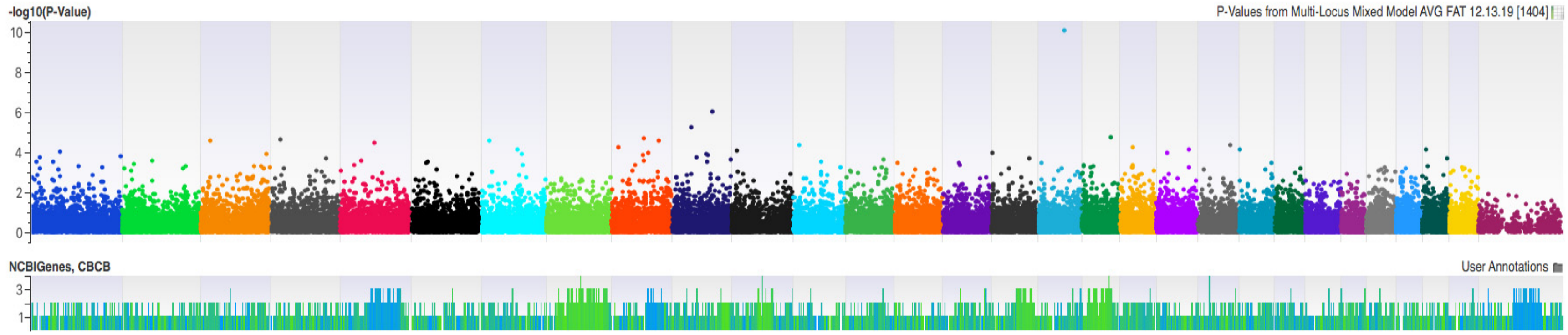


Supplementary Figure S6. Manhattan plot for average 12th rib fat (BF). Markers above –log10(*p*-value) of 5 x $10^{-8}$ are genome-wide association significant markers. Vertical clusters of markers are also of interest as they are indicating suggestive QTL in those regions.

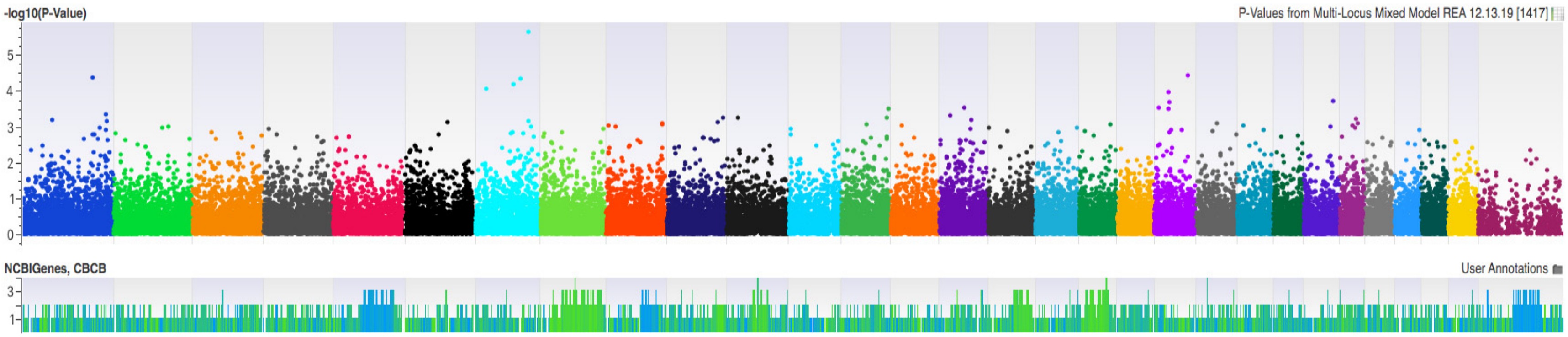


Supplementary Figure S7. Manhattan plot for rib eye area (REA). Markers above –log10(*p*-value) of 5 x $10^{-8}$ are genome-wide association significant markers. Vertical clusters of markers are also of interest as they are indicating suggestive QTL in those regions.

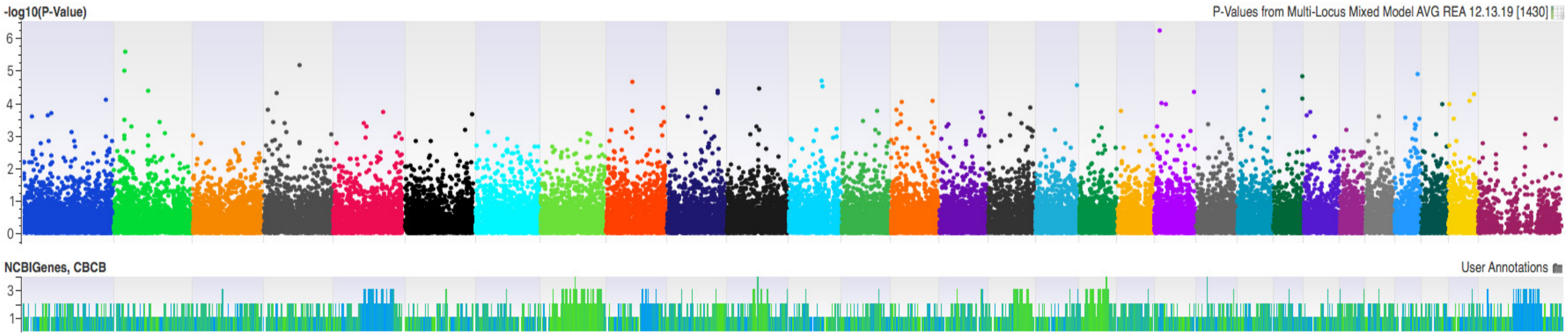


Supplementary Figure S8. Manhattan plot for average rib eye area (REA). Markers above –log10(*p*-value) of 5 x $10^{-8}$ are genome-wide association significant markers. Vertical clusters of markers are also of interest as they are indicating suggestive QTL in those regions.
